# Genomic medicine in the Middle East

**DOI:** 10.1186/s13073-021-01003-9

**Published:** 2021-11-23

**Authors:** Ahmad N. Abou Tayoun, Khalid A. Fakhro, Alawi Alsheikh-Ali, Fowzan S. Alkuraya

**Affiliations:** 1 Al Jalila Genomics Center, Al Jalila Children’s Hospital, Dubai, United Arab Emirates; 2 Center for Genomic Discovery, Mohammed Bin Rashid University of Medicine and Health Sciences, Dubai, United Arab Emirates; 3 Department of Human Genetics, Sidra Medicine, Doha, Qatar; 4 Department of Genomic Medicine, Weill Cornell Medical College, Doha, Qatar; 5 Dubai Health Authority, Dubai, United Arab Emirates; 6 College of Medicine, Mohammed Bin Rashid University of Medicine and Health Sciences, Dubai, United Arab Emirates; 7 Departement of Translational Genomics, Center for Genomic Medicine, King Faisal Specialist Hospital and Research Center, Riyadh, Saudi Arabia

## Abstract

We discuss the current state of genomic medicine in Arab countries of the Middle East, a region with outsized contribution to Mendelian genetics due to inbreeding yet has poor representation in global variome datasets. We focus on genomic testing, clinical genetics, and genetic counseling services along with associated training and research programs. Finally, we highlight opportunities for improvement in genomic medicine services in this region.

## Background

With the completion of the first telomere-to-telomere human genome [1], the continuous proliferation of databases capturing genetic variation across different ancestries, the continuous annotation of disease-related genes, and the accelerated development of nucleic acid-based therapeutics, it has become clear that incorporation of genomics into almost all aspects of medical practice is inescapable to guarantee best healthcare delivery. Building on these advancements, the National Human Genome Research Institute crafted bold predictions emphasizing that, in the next 10 years, “the regular use of genomic information will have transitioned from boutique to mainstream in all clinical settings, making genomic testing as routine as complete blood counts” [2].

Alignment with such predictions and integration of genomic medicine into healthcare provision require significant and strategic investments in building the infrastructure necessary for efficient utilization of genomics in diagnostic and screening settings, in “sick” and “healthy” populations, and across several clinical applications (e.g., cancer, rare diseases, pharmacogenetics, and prenatal genetics). Furthermore, to guarantee long-term sustainability, genomic medicine programs cannot be disentangled from robust research agendas and specialized training programs. Although successful implementation of genomic medicine is evident in the USA and Europe, a closer look at such resources in other geographical regions is highly warranted given that diversification of genomics efforts is crucial to propel the whole field forward.

## Current state of genomics in the Middle East

With a population of over 400 million, a tradition of consanguineous marriages, and large family structures, Arabs of the Middle East face a relatively high burden of Mendelian recessive disorders [3]. Arguably, effective implementation of genomic medicine in the Middle East can set the clearest example for (1) better preventive medicine through premarital, preconception, prenatal, and/or newborn genetic screening; (2) earlier diagnostics and timely intervention; and (3) improved novel gene discovery through research programs focusing on undiagnosed families with suspected Mendelian diseases.

Unfortunately, however, genetic services in the Middle East remain relatively fragmented, generally motivated by individual interests, and often lack a centralized or a population-based national strategy. More recently, some trends have begun to reverse as detailed below.

### Genomic diagnostic and screening services

Recognizing the genetic disease burden, several Middle Eastern countries began implementing national newborn screening programs since the early 2000s to identify affected newborns for timely intervention. Such programs, while important, only screened for a handful of inborn errors of metabolism, hearing loss, and/or hemoglobinopathies [4]. More recently, given their high prevalence, premarital screening for hemoglobinopathies (thalassemia and sickle cell disease) has been mandated in many countries including the Kingdom of Saudi Arabia (KSA), Bahrain, Iraq, Qatar, and the UAE. Consequently, the prevalence of sickle cell disease in Bahrain, for example, has been significantly reduced, though in general couples still maintain the autonomy to proceed with a marriage despite the risks for disease outcomes [5]. Furthermore, population genetic screening, in the form of limited or expanded gene panels, is still lacking in the Middle East.

Cytogenetic services, mostly based on traditional karyotyping and FISH, have become widely available in major hospitals within the region. Several centers have also started moving into molecular karyotyping or chromosomal microarrays.

Perhaps the most prominent gap in the region is the lack of adequate clinical genomic-sequencing facilities. The development of cost-effective, next generation sequencing (NGS) technology has shifted the clinical genetics testing paradigm towards comprehensive sequencing, in the form of large constitutional or somatic gene panels and whole exome sequencing, especially for highly heterogenous genetic disorders. This technology is also the backbone of non-invasive prenatal testing (NIPT) which screens for trisomies in high-risk pregnancies. The demand for NGS-based testing currently outstrips local capacity, and several Middle Eastern countries rely on reference laboratories mainly in Europe and the USA to meet this demand. Currently, we are aware of only 16 laboratories in the Middle East which are accredited by the College of American Pathologists to perform molecular genetic testing. Assuming all those laboratories perform NGS-based testing (which will be an overestimate), this figure still illustrates a severe shortage of such services on a per capita basis in the region when compared to a relatively similar sized population in the USA, which houses hundreds of such NGS laboratories.

The main reason for this gap lies in the fact that the Middle East has traditionally faced brain-drain of talent relocating overseas in search for professional growth. This has led to a shortage of well-trained personnel—especially clinical molecular geneticists, bioinformaticians, computational biologists, and genomic analysts—needed to establish and operate the newest platforms for highly complex genomic testing, which not only requires complex wet bench processing, but also the manipulation of large datasets for clinical interpretation and reporting in a clinically compliant environment. A report by the American Board of Medical Genetics and Genomics (ABMGG) in 2018 [6] showed that there were 11 board-certified clinical molecular geneticists practicing in the Middle East (0.02 per million population) as opposed to 632 in the USA (~ 2 per million population) at that time.

To boost local capacity in that area, several countries including UAE, Qatar, KSA, and Lebanon have taken important steps towards establishing clinical genomic sequencing facilities and local expertise. The advantage of building such capacity goes beyond the ability to perform testing locally, in a cost-effective manner and with a faster turnaround time, into the ability to embed training programs that prepare local trainees for all aspects of genomic medicine, as there are currently very few training programs in the region. Furthermore, as more clinical sequencing tests are performed locally, it will gradually become easier to establish disease databases cataloging sequence variants specific to this population, which will in turn refine the clinical interpretation process and inform carrier screening programs in this region.

### Genetic counseling services

Appropriate pre- and post-test genetic counseling is essential for families undergoing diagnostic or screening (premarital, prenatal, newborn) testing. Currently, there is a significant shortage in Arabic-speaking genetic counselors who are familiar with local customs and are well-trained to support genetic counseling for families in screening and diagnostic settings. Based on a recent report, we estimate the presence of around 40 genetic counselors in the Arab region (0.1 per million population) which is at least 20-fold less than the UK (5 per million population) and the USA (12 per million population) [7].

To start addressing this gap, some countries are now establishing training programs in genetic counseling. There are at least two master’s level genetic counseling training programs in Qatar and KSA, and more programs are likely to be launched soon in other countries. However, such programs still face a steep road ahead as they need to grow significantly to train enough local talent and to meet the significant genetic counseling demand in this growing population.

### Genetics clinics

Clinical genetics in the region was mainly driven by grassroot efforts of a few individuals whose background was primarily in pediatrics. The large demand created by the high rate of autosomal recessive Mendelian diseases [3], and early identification of positive cases through newborn or premarital screening, led to the expansion of clinical genetics services, more rapidly in some countries than others.

However, according to a 2018 ABMGG report [6], 1376 board-certified clinical and biochemical geneticists were practicing in the USA which is equivalent to ~ 4 geneticists per million population. While very few countries in the region might be close to such ratio, the majority remain very far off leaving a severe gap amidst the relatively larger demand. Local training programs in medical genetics, some in the form of fellowships after pediatric residency, have been launched in a few countries like Egypt, KSA, and Qatar. Additional training programs will be essential to prepare the next generation of geneticists across the Middle East and to meet this population needs.

### Genomic medicine research

The recent proliferation of NGS platforms has transformed the landscape of genetic research from very few centers with expertise in positional gene mapping approaches to more players from major academic centers in the region. For example, a successful gene discovery program largely focused on Mendelian recessive disorders in KSA identified hundreds of novel gene-disease associations this far and has been the subject of several profiles and awards [8]. Scientists in Qatar have also began implementing NGS at the point of care and have contributed several key population genetics findings [9], while the UAE has recently launched a genomics diagnostics center (Al Jalila Genomics Center, www.genomics.ae) along with a Center for Genomic Discovery (https://www.mbru.ac.ae/research/the-center-for-genomic-discovery/) for novel gene discovery in pediatric diseases. Those programs will not only benefit the local population in terms of enhancing diagnostics and recurrence risk assessments, but will also complement the functional annotation and our understanding of the human genome in general [10].

However, there is still a need to diversify the research portfolio to further enhance Mendelian gene discoveries and to embrace common diseases given the advancements in integrating polygenic risk scores in clinical medicine. Local efforts to address the population stratification and its impact on the transferability of polygenic risk scores developed in other mainly European populations are underway.

## Moving forward and concluding remarks

The path forward is clear: genomic medicine is a national healthcare priority for several countries in the Middle East. There have been several well-organized, national-level initiatives recently in countries such as Egypt, Qatar, KSA, and UAE to enhance the integration of genomics into healthcare, including large-scale population genomic projects, the establishment of local training and research programs, and the expansion of genetic services. Nonetheless, significant national investments and alignment across borders are still needed to integrate existing genetics programs with one another on a regional level, and to establish new ones in other countries within the Middle East.
